# Targeted optical fluorescence imaging: a meta-narrative review and future perspectives

**DOI:** 10.1007/s00259-021-05504-y

**Published:** 2021-10-11

**Authors:** H. M. Schouw, L. A. Huisman, Y. F. Janssen, R. H. J. A. Slart, R. J. H. Borra, A. T. M. Willemsen, A. H. Brouwers, J. M. van Dijl, R. A. Dierckx, G. M. van Dam, W. Szymanski, H. H. Boersma, S. Kruijff

**Affiliations:** 1 Department of Surgery, University of Groningen, University Medical Centre Groningen, Groningen, The Netherlands; 2 Department of Nuclear Medicine and Molecular Imaging, University of Groningen, University Medical Centre Groningen, Groningen, The Netherlands; 3 Department of Biomedical Photonic Imaging, Faculty of Science and Technology, University of Twente, Enschede, The Netherlands; 4 Department of Radiology, University of Groningen, University Medical Centre Groningen, Groningen, The Netherlands; 5 Department of Medical Microbiology, University of Groningen, University Medical Centre Groningen, Groningen, The Netherlands; 6 Department of Diagnostic Sciences, Ghent University Faculty of Medicine and Health Sciences, Gent, Belgium; 7 AxelaRx/TRACER Europe BV, Groningen, The Netherlands; 8 Department of Clinical Pharmacy and Pharmacology, University of Groningen, University Medical Centre of Groningen, Groningen, The Netherlands

**Keywords:** Optical fluorescence imaging, Fluorescence, Oncology, Cardiovascular disease, Infectious disease

## Abstract

**Purpose:**

The aim of this review is to give an overview of the current status of targeted optical fluorescence imaging in the field of oncology, cardiovascular, infectious and inflammatory diseases to further promote clinical translation.

**Methods:**

A meta-narrative approach was taken to systematically describe the relevant literature. Consecutively, each field was assigned a developmental stage regarding the clinical implementation of optical fluorescence imaging.

**Results:**

Optical fluorescence imaging is leaning towards clinical implementation in gastrointestinal and head and neck cancers, closely followed by pulmonary, neuro, breast and gynaecological oncology. In cardiovascular and infectious disease, optical imaging is in a less advanced/proof of concept stage.

**Conclusion:**

Targeted optical fluorescence imaging is rapidly evolving and expanding into the clinic, especially in the field of oncology. However, the imaging modality still has to overcome some major challenges before it can be part of the standard of care in the clinic, such as the provision of pivotal trial data. Intensive multidisciplinary (pre-)clinical joined forces are essential to overcome the delivery of such compelling phase III registration trial data and subsequent regulatory approval and reimbursement hurdles to advance clinical implementation of targeted optical fluorescence imaging as part of standard practice.

**Supplementary Information:**

The online version contains supplementary material available at 10.1007/s00259-021-05504-y.

## Introduction

During the past decades, fluorescence-based optical imaging progressed from microscopy and animal studies to human studies and, today, it is finally entering clinical practice in several disease areas. The imaging technology is based on electromagnetic radiation in the range of energies that correspond to the ultraviolet–visible-near infrared (NIR) spectrum of light [1–4]. This distinguishes optical, or more specifically defined in this review as fluorescence imaging, from other imaging techniques using strong magnetic fields combined with radio wave frequencies such as magnetic resonance imaging (MRI) or ionizing X-ray and gamma radiation such as planar X-ray imaging, computed tomography (CT), positron emission tomography (PET) and single photon emission computed tomography (SPECT) [5]. Optical fluorescence imaging approaches have been implemented in various biomedical and clinical applications, including microscopy, endoscopy and image-guided surgery [1, 2]. Detection systems used for optical fluorescence imaging are relatively inexpensive and portable, compared to other imaging modalities. This, in combination with the minimally invasive character of optical fluorescence imaging, renders it a highly promising diagnostic bedside technique [1]. The main current limitation of optical fluorescence imaging stems from the opacity of the human body for the visible light and the resulting limited light penetration depth. The use of NIR fluorophores and dyes as optical fluorescence imaging labels can provide at least a partial solution to overcome the depth penetration limitation since they fluoresce at wavelengths which exhibit the least absorption of light by haemoglobin, the most abundant light absorber in the human body, and show diminished scattering. This combined with the lowest presence of autofluorescence results in depth penetration of light in the range of centimetres through soft tissues [6, 7].

Just like nuclear imaging techniques, fluorescent imaging enables visualization of biological processes through the use of targeted or non-targeted tracers. Non-targeted tracers can accumulate passively in for example tumorous processes or aid in visualizing tissue perfusion [8–10]. Currently, there is ample experience with non-targeted tracers, such as indocyanine green (ICG) [11]. For example, ICG recently demonstrated exceptional results in imaging of metastasis of sarcomas and pancreatic and lung cancer [12–14]. Additionally, the detection of target tissue autofluorescence and spectroscopic imaging methods are gaining terrain. The advantage of the latter methods is that the respective tracers do not need to be registered or approved [15, 16]. Nonetheless, the demand for increased specificity has resulted in a shift towards the development of targeted fluorescent tracers [17]. These targeted tracers are composed of a carrier molecule (e.g. an antibody, peptide or small molecule) with a fluorescent probe attached to it, directed at a specific disease biomarker [18]. Clinical translation of targeted and non-targeted fluorescent imaging is often challenged by chemical properties of the dye and therefore also its photophysical properties, tracer conjugation difficulties, tracer synthesis steps, toxicology and ultimately pharmacodynamics and pharmacokinetics in vivo. Hence, a multidisciplinary research team including chemists, pharmacists, engineers and clinicians is a prerequisite for the development of successful optical fluorescence imaging agents for clinical applications [19–22].

To provide a comprehensive overview of the current state of the field and to identify the potential pitfalls for targeted fluorescence imaging, we have assembled the present meta-narrative review. This review is enriched with possible directions for the future development of clinical fluorescence imaging towards bedside implementation of this technique. With this review, we ultimately aim to inspire readers to further explore and expand the clinical translation of optical fluorescence imaging techniques to the benefit of patients suffering from oncological, cardiovascular, infectious and inflammatory diseases.

## Background of optical imaging techniques

The first reference to fluorescence dates back to 1845, when John Herschel reported the blue colouration of tonic water, a quinine solution, under UV light [23]. Subsequently, in 1852, the term “fluorescence” was coined [24]. The phenomenon of fluorescence became widely applicable after fluorescent probes or fluorophores were introduced to study molecular processes [25]. Since then, multiple optical techniques have emerged, especially in the second half of the twentieth century. In particular, fluorescein and ICG have become widely known for their in vivo applications. Fluorescein is used in ophthalmology and ICG is used to visualize and quantify blood flow, in, for example liver and biliary anatomy and evaluation of skin-flap viability [11, 26].

**Fig. 1:**
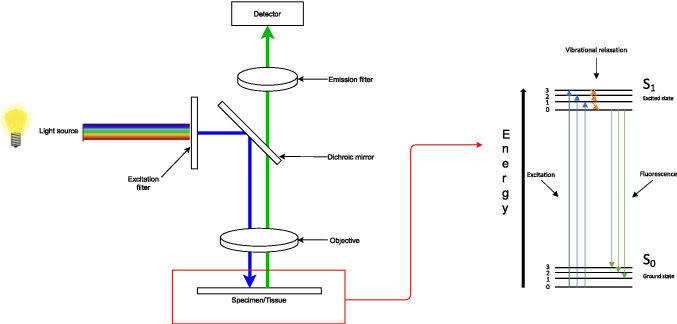
Representation of the concept of fluorescent imaging. Light from a light source, filtered by an excitation filter is deflected by a dichroic mirror before it hits the (tissue) specimen. Consequently, fluorophores in the specimen absorb photons, which results in promotion to an excited state. After losing a part of their energy in the process of vibrational relaxation, the molecule falls back to the ground state by emitting a photon of a lower energy and a longer wavelength. The emitted signal passes through an objective and an emission filter before it hits a detector

Fluorophores are molecules that absorb photons, which trigger an excited state of the fluorophore’s electrons. After losing a part of their energy in the process of vibrational relaxation, the molecule’s electrons fall back to the ground state by emitting a photon of a lower energy and thus a longer wavelength. This difference in energies between the energies of absorbed and emitted light is called the Stokes shift [24]. Depending on the wavelength of the emitted light, it is either visible by the human eye (380–700 nm) or detectable by (near) infrared cameras (> 700 nm) in the so-called near-infrared fluorescence (NIRF) imaging. Repeated excitation of fluorophores is possible, as the molecules relax from their excited state to their ground state in the span of nanoseconds, rendering them again readily available for photon absorption (Fig. 1) [27]. The principle of NIRF imaging is also applicable to endomicroscopy through integrating light sources and cameras in endoscopes [28].

A different approach to optical fluorescence imaging is the so-called photoacoustic molecular imaging (PAMI), of which multispectral optoacoustic tomography (MSOT) is a subtype. It relies on the light-induced excitation of a dye molecule, similar to what happens in fluorescence optical imaging. However, a photoacoustic imaging dye returns to the ground state through a thermal relaxation process, which leads to local heating of the tissue and concurrent thermoelastic expansion. In turn, this expansion produces acoustic waves that can be detected using an ultrasound transducer [29, 30].

**Fig. 2:**
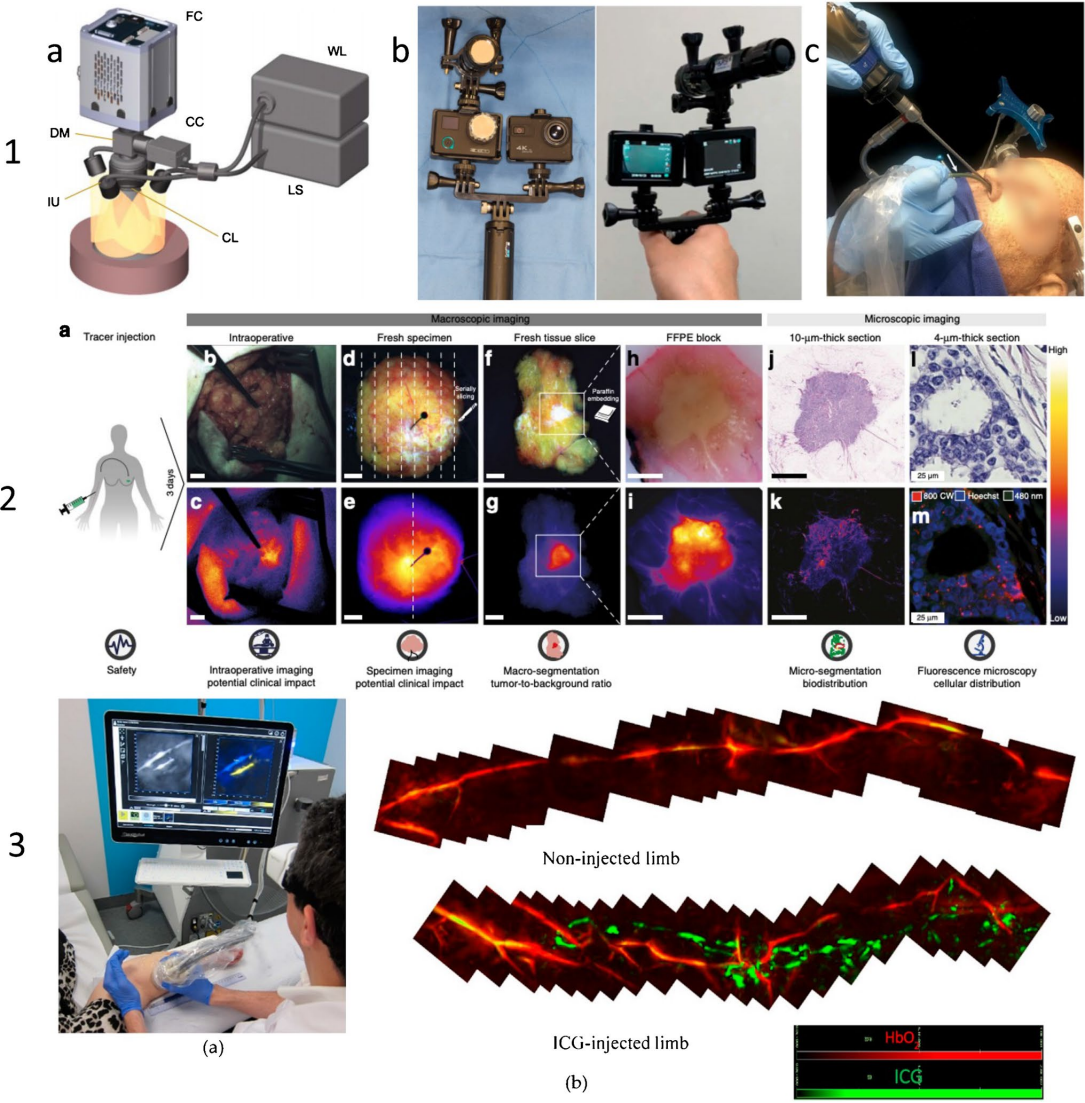
**1**a Typical composite camera system using a highly sensitive fluorescence camera (FC) to collect fluorescence images and a colour camera (CC) to collect white-light images through a dichroic mirror (DM) and a common lens (CL). Different light sources may be used for white-light excitation (WL) and fluorescence excitation using a laser source (LS) and common illumination unit (IU). Reprinted by permission from Springer Nature Customer Service Centre GmbH, Springer Nature, Nature Photonics, Tackling standardization in fluorescence molecular imaging, Koch et al. [31] Copyright (2018). **1**b Front and back of ICG-NIRF prototype modified action camera with 7.2-mm lens, modified action camera with 7.2-mm lens and bandpass filter and modified LED light with bandpass filter. Reproduced from Yang et al. [32] J. Clin. Med. Copyright 2021 MDPI, Basel, Switzerland. **1**c Position of confocal laser endomicroscopy (CLE) scanning probe in an endonasal transsphenoidal approach (arrow points to the CLE probe). Reproduced from Belykh et al. [33] J. Clin. Med. Copyright 2020 MDPI, Basel, Switzerland. **2**a Intravenous administration of bevacizumab-800CW 3 days prior to surgery. **2**b, c Colour image and corresponding fluorescence image obtained in vivo during surgery to determine potential clinical value. **2**d, e Imaging of the fresh surgical specimen, followed by serially slicing. **2**f, g Imaging of the fresh tissue slices to determine tumour-to-background ratio based on macro-segmentation, followed by paraffin embedding. **2**h, i Imaging of formalin-fixed paraffin-embedded (FFPE) blocks to determine heterogeneity of tracer uptake within a tumour. **2**j, k Imaging of 10-μm-thick tissue sections for microsegmentation to reveal microscopic biodistribution and correlation with fluorescence signals from the macroscopic to microscopic level. **2**l,m Fluorescence microscopy to determine tracer distribution on a cellular level. Scale bars represent 1 cm, in l, m the scale bar represents 25 μm. Reproduced from Koller et al. [34] Nature Communications Copyright 2018, Springer Nature. **3**a Bedside multispectral optoacoustic tomography (MSOT) examination. **3**b In the ICG injected limb both lymphatic (green) and blood vessels (red) were detected while in the non-injected limb, only blood vessels (red) could be detected. Reproduced from Giacalone et al. [35] J. Clin. Med. Copyright [2020], MDPI, Basel, Switzerland

The technical backgrounds of NIRF, fluorescence endomicroscopy and PAMI/MSOT are summarized in Table 1. Examples of imaging equipment are presented in Fig. 2.

**Table 1:** Overview of utilized optical fluorescence imaging techniques and investigated fluorophores as described by the included articles

Imaging technique	Description technique	Abbreviation
Near-infrared fluorescence imaging	Sensitive cameras detect fluorescent signals in the target tissue and construct 2D images, both in and ex vivo. These signals can be enhanced by the administration of fluorophores	NIRF imaging
Multispectral optoacoustic tomography	Delivers short laser pulses to target tissue and/or fluorophores, producing heat and thereby expanding the target tissue, giving rise to ultrasound waves. These signals can be converted to 3D images	MSOT
Optical endomicroscopy	Enables imaging of tissue histology in situ, allowing for cross-sectional images on the micron scale through the use of endoscopes, catheters, laparoscopes and needles	OEM

Fluorophore/probe	Excitation wavelength (nm)	Emission wavelength (nm)
BM104	680–685	700–710
Cy5.5	650–683	700–720
Fluorescein (Isothiocyanate)	475–490	510–520
ICG	740–800	800–860
IRDye800CW	770–780	790–798
NBD	460–480	520–550
S0456	774–776	794–796
ZW800-1	710–806	780–900
MMPSense680	670–690	690–710

Fluorophores and dyes thus allow for real-time visualization of structures, both macro- and microscopically. Additionally, the development of specific cross-linking techniques for the fluorescent labelling of proteins, antibodies and small molecules has strongly improved the specificity of tracers towards targeting tissues in for example receptor targeting [18]. In fact, multiple fluorophores may be utilized simultaneously in one and the same clinical application by combining molecules with different excitation and emission spectra, as was recently described in research regarding penile cancer where a Cy5 tracer targeting the MET receptor was simultaneously used with ICG [36].

## Methods

### Study design and search strategy

To provide an overview of the currently available targeted optical agents and their (potential) clinical implementation, a meta-narrative approach was chosen [37]. Furthermore, this paper was structured following the RAMESES (Realist And Meta-narrative Evidence Syntheses: Evolving Standards) publication standards [38]. A systematic search was performed using the PubMed database. Three distinct searches were performed with keywords related to “Optical fluorescence imaging” as constant (see Supplement 1 for further information).

### Inclusion criteria and selection process

**Fig. 3:**
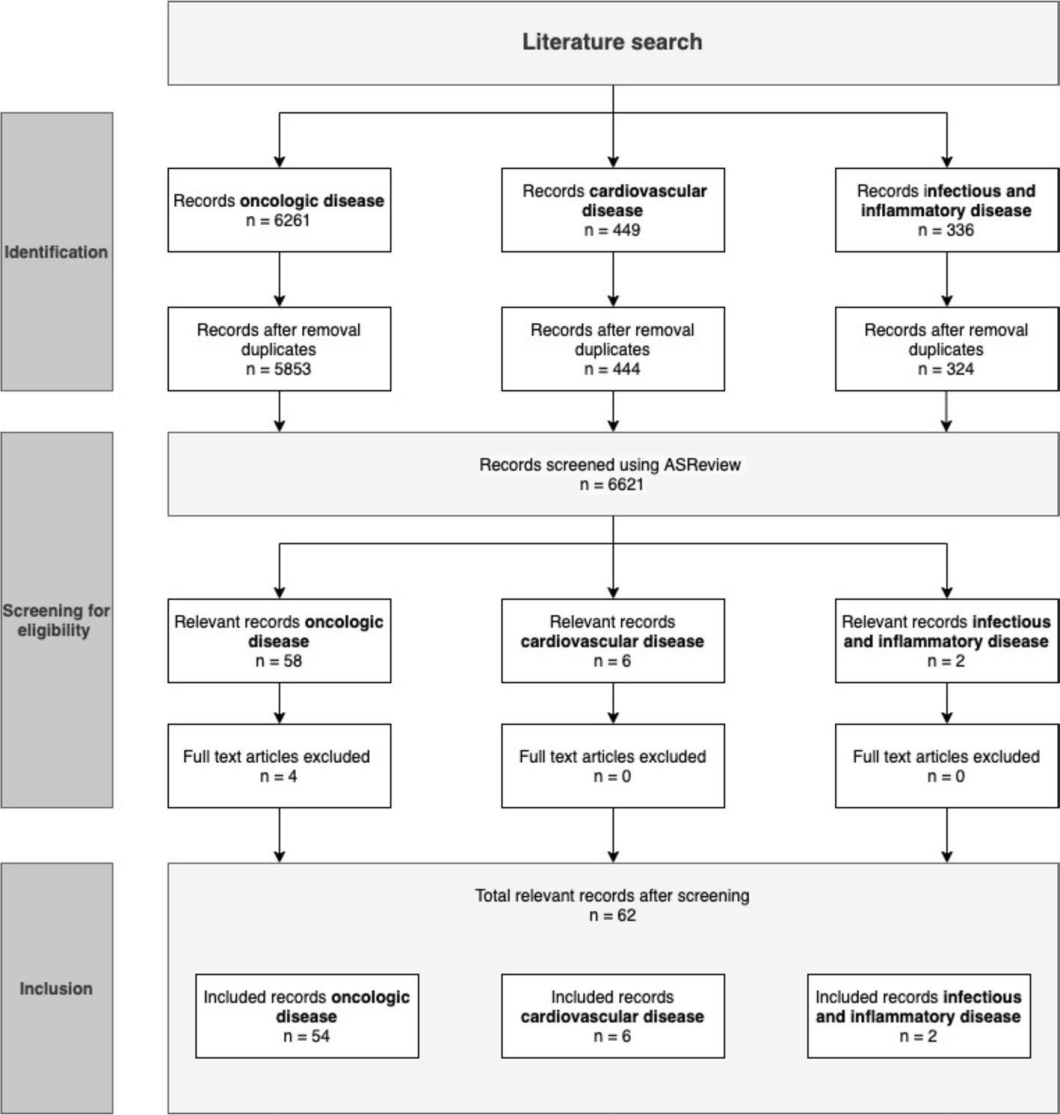
Flow diagram of literature search

Original human clinical studies that investigated a targeted optical tracer in the field of oncologic, cardiovascular, infectious and inflammatory diseases were included in this review. All publications that did not meet these requirements were excluded. Furthermore, all non-English publications, papers without complete abstracts and unobtainable paper copies were also excluded (Fig. 3).

Because of the large number of resulting works, titles and abstracts were screened and assessed for eligibility using the artificial intelligence program ASReview version v0.16 [39]. This software is based on machine learning algorithms that includes active learning and interaction with the researcher. It minimizes human error during the screening process and, consequently, increases the screening efficiency [39, 40]. Inter-rater reliability was established by two independent researchers. We calculated an inter-rater reliability of 97%. Furthermore, the guideline produced by ASReview was followed to screen an additional 25% of the total number of search results after the last relevant marked article, to minimize the risk of missing additional relevant articles [40].

The relevant articles screened with ASReview were read in full text. References of articles were also scanned and the duplicates were removed. An overview of the literature selection process is presented in Fig. 3. The final eligible articles were organized using Mendeley’s reference management software [41].

### Data extraction and analysis

All relevant journal metadata were extracted from the included papers (Table 2). The latter were grouped by investigated disease type and investigated optical tracer.

**Table 2:**
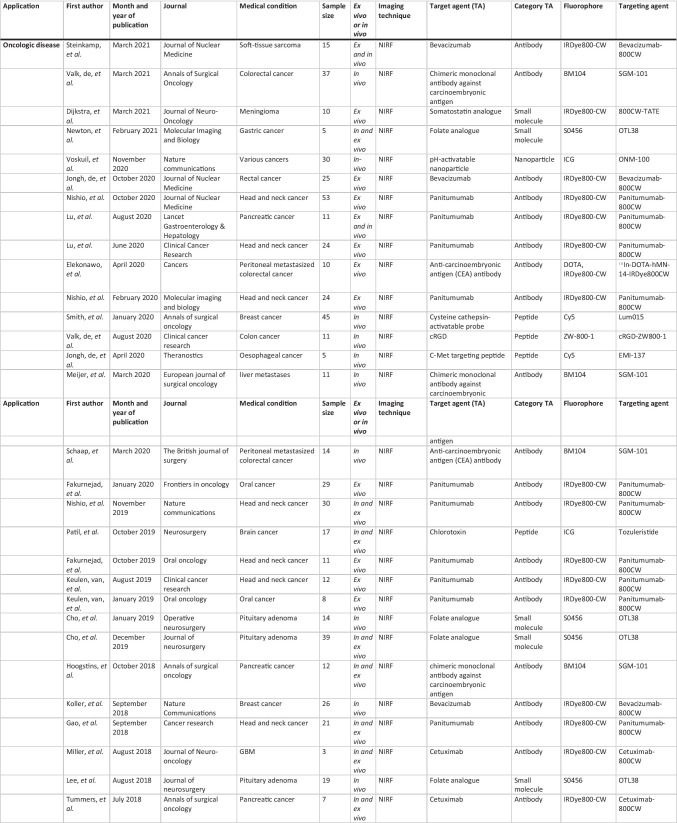

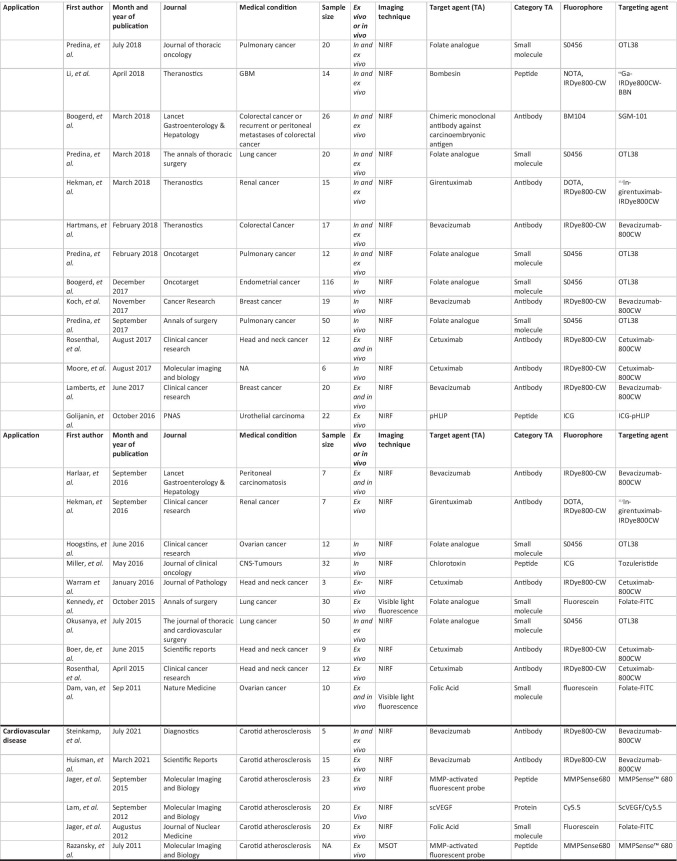

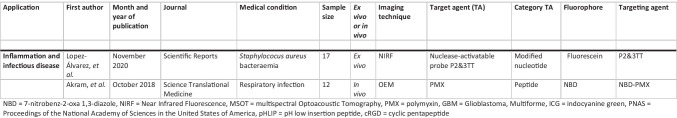
Overview included articles per general category

**Fig. 4:**
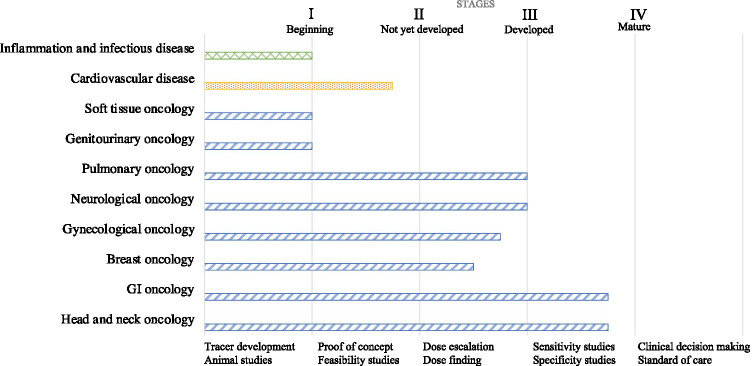
The different fields of optical fluorescence imaging and their corresponding stages of clinical implementation

Fields of interest were assigned to a developmental stage based on the combined data per field of interest, study design (e.g. ex vivo, in vivo, both), analysis (e.g. sensitivity and specificity) and clinical decision-making (e.g. alteration of (surgical) treatment plan). In this process, four different stages of maturity emerged: stage I, beginning stage (feasibility studies); stage II, not yet developed (dose-escalation studies); stage III, developed (sensitivity and specificity studies); and stage IV, mature, standard of care (Fig. 4).

## Results

### Selected articles and study characteristics

The search produced 6261 peer-reviewed articles, related to targeted and non-targeted optical fluorescence imaging, to be screened with ASReview: 5853 involved oncological disease, 449 cardiovascular disease and 336 infectious and inflammatory diseases. Finally, 54, 6 and 2, of the respective papers met the inclusion criteria (see Table 2 for an overview of the included studies and Fig. 3 for the selection process). The high exclusion percentage is explained by the large amount of non-targeted optical imaging studies and animal studies.

In the following paragraphs, results are summarized based on pathologies within the major clinical domains and the optical imaging methodologies applied. Each paragraph concludes with the assigned developmental stage.

### Optical fluorescence imaging in oncology

#### Head and neck oncology

The therapeutic antibodies panitumumab and cetuximab, targeted against Epidermal Growth Factor Receptor (EGFR), are used for the treatment of head neck squamous cell carcinoma (HNSCC). Their high specificity for EGFR makes them ideal candidates for targeted optical fluorescence imaging. Cetuximab, conjugated to the GMP-produced NIR fluorescent dye NHS-IRDye800CW (800CW) (Table 1), has been used for precise peri-operative identification of tumour tissue during surgery, back-table imaging and for combining standard histopathology with in vivo fluorescence immunohistochemistry [42, 43]. Compared to the gold standard of histological assessment, fluorescence-guided pathology showed a sensitivity and specificity of 91.0% and 85.0%, respectively [44]. In a later phase, intraoperative fluorescence imaging using cetuximab-800CW was able to identify tumour-positive and -negative lymph nodes with a sensitivity and specificity of 97.2% and 92.7%, respectively. Fluorescence imaging reliably detected cancer and altered tumour staging in 8.1% of lymph nodes that were considered false positive by histopathology. Furthermore, a pre-operative unlabelled dose of cetuximab showed significant improvement of intraoperative performance. This was likely due to increased off-target receptor occupancy by the unlabelled cetuximab, resulting in higher tumour uptake, an effect which is also seen in radioimmunotherapy [45–47].

Similar studies targeting EGFR have been performed with panitumumab-800CW. Several clinical studies showed around 89% sensitivity and a negative predictive value (NPV) > 90% during surgical specimen tumour mapping. Similarly, panitumumab-has aided in tumour detection and ex vivo mapping of margins with a sensitivity of 95%, making it a potential valuable margin-evaluation tool to accelerate intraoperative decision-making by the attending surgeon. Furthermore, panitimumab-800CW allowed distinction between low-grade and high-grade dysplasia in fluorescence histopathology [48–51]. Additionally, ex vivo optical fluorescent specimen imaging with panitimumab-800CW was proposed to reduce sampling errors in tissue selection for frozen section analysis and facilitated the surgeon’s orientation to which areas needed to be sampled [52]. Panitumumab-800CW enhanced the workflow by allowing the discrimination of metastatic and benign lymph nodes, resulting in a 90% reduction of lymph nodes that have to be pathologically and histologically examined [53, 54]. Furthermore, it was shown that pre-operative contrast-enhanced MRI is capable of predicting intra-tumoral distribution and accumulation of panitumumab-800CW and can therefore assist in selection of patients suitable for intraoperative optical fluorescent imaging with panitimumab-800CW [55].

Recently, PAMI after administration of panitumumab-800CW appeared to have the potential to improve the diagnosis of lymph node metastasis by providing enhanced imaging resolution at an increased depth in human ex vivo neck specimens from HNSCC patients [56].

Another novel technique is smart-activated optical fluorescent imaging. In summary, a fluorescent-silent (i.e. quenched) tracer is administered topically or systemically, and activation by a biological disease results in an un-quenched state of the fluorophore, and thus, the tracer is in its “on” state and able to be excited resulting in subsequent emission of light [57]. ONM-100 is a smart activatable pH-sensitive amphiphilic polymer nanoparticle or micelle, consisting of multiple quenched ICG molecules within the particle (Table 1). These micelles, tuned for a pre-defined pH setpoint, rapidly disintegrate in an acidic tumour environment, creating un-quenching of the ICG molecules and thus a fluorescent signal upon excitation. In a recently published phase I clinical study, ONM-100 allowed the detection of tumour-positive resection margins intraoperatively, as well as ex vivo, with a sensitivity and specificity of 100% and 57% respectively [58].

Since targeted optical fluorescence imaging in head and neck cancer facilitates workflow optimization and, in some instances improved tumour staging, this field was classified as presently in end-stage III (Fig. 4).

#### Gastrointestinal oncology

Bevacizumab conjugated to NHS-IRDye800CW targets the soluble ligand of vascular endothelial growth factor A (VEGF-A). In peritoneal carcinomatosis of colorectal origin, imaging with bevacizumab-800CW was able to reveal additional histologically confirmed tumour tissue, which was initially not detected by oncological surgeons by visual and manual inspection alone. Eight out of 79 samples were false-negative lesions and no false positives were reported, as confirmed by final histological analysis [59]. Furthermore, bevacizumab-800CW-guided fluorescence endoscopy and back-table margin imaging in patients with locally advanced rectal cancer showed a higher sensitivity, specificity and accuracy compared to MRI [60, 61].

Fluorescence endoscopy with the same tracer in Barrett’s oesophagus and familial adenomatous polyposis (FAP) patients resulted in sufficient tumour-to-background ratios (TBRs) for lesions smaller than 3 mm. Furthermore, fluorescence imaging identified additional lesions missed by simple white-light imaging. This suggests that fluorescence endoscopy can aid in early diagnosis of (pre)malignant tissue [62, 63]. Additionally, fluorescence endoscopy imaging with a small peptide conjugated to a Cy5 fluorescent dye (EMI-137), targeting the c-Met receptor, was investigated in vivo in Barrett’s oesophagus patients. Here, a 100% sensitivity was reached. Histologically, three additional lesions identified with white-light endoscopy displayed a low expression of c-Met and were therefore not detected by fluorescence imaging [64].

In pancreatic cancer patients, with tumours known for impermeable tissue characteristics as a result of the inflammatory desmoplastic tissue reaction, both panitumumab-800CW and cetuximab-800CW have been investigated [65]. A feasibility study with panitumumab-800CW demonstrated that it was possible to visualize primary tumours, lymph node metastasis and small (< 2 mm) peritoneal metastasis, despite the size of the antibody-fluorescent dye conjugate of ~ 150 kD which might hamper tissue-specific targeting due to the aforementioned desmoplastic reaction. For primary tumour detection, panitumumab-800CW showed a 90.3% sensitivity and 74.5% specificity, compared to immunohistochemistry stains, while cetuximab-800CW showed 96.1% sensitivity and a 67.0% specificity [66, 67]. Pancreatic cancer was also visualized with SGM-101, an antibody specific for the carcinoembryonic antigen (CEA) labelled with fluorescent BM104 (Table 1). Two clinical studies showed that both the primary tumour and metastatic lesions could be visualized with 89% accuracy [68, 69].

SGM-101 was also evaluated in patients with primary colorectal cancer (CRC) and, if present, its peritoneal metastases. Imaging of fluorescent and suspected lesions resulted in 98% sensitivity and 62% specificity. Intraoperative imaging altered care in 24–34% of patients. Lastly, 44% of the histologically proven malignant lesions were only identified with fluorescence imaging and missed by clinical assessment through routine visual and manual inspection [70–72]. One study investigated the hybrid tracer ^111^In and 800CW-labelled CEA-targeting antibody labetuzumab (^111^In-DOTA-hMN-14-800CW). Ex vivo incubation of CRC lesions showed a fivefold increase in median fluorescent and autoradiography intensities as compared to surrounding tissue. Deeper tissue sections demonstrated less tracer uptake, potentially missing deeper seated target tissue in vivo [73]. The fluorescently labelled peptide cRGD-ZW800-1 (Table 1) was further investigated in targeting a variety of integrins in CRC patients and healthy volunteers. In the latter group, healing wounds showed fluorescence caused by cRGD-ZW800-1, which could potentially lead to overtreatment when translated to intraoperative decision-making [74].

In gastric cancer patients, the NIR tracer OTL38 (folate conjugated to the fluorophore S0456) (Table 1) was investigated as a tracer targeting folate receptor-α overexpression in gastric cancer. Intraoperatively, fluorescence imaging could be detected extraluminally through the stomach wall. Clinically suspect lesions that did not display fluorescence were sampled and showed indeed benign polyps and benign liver tissue [75].

Furthermore, several ongoing trials are pending on the abovementioned tracers bevacizumab-800CW, cetuximab-800CW and SGM-101 (for example NCT03620292, NCT04638036 and NCT04642924). Lastly, the novel tracer vedolizumab-800CW is being studied in IBD patients to predict the treatment response and to elucidate the targeting agent’s mechanism of action (NCT04112212).

Altogether, multiple studies demonstrated that the surgical plan was altered or could have been altered intraoperatively, which emphasizes the added value of clinical fluorescence imaging and the potential of fluorescence-guided surgery. For this reason, optical fluorescence imaging in gastrointestinal cancers has reached stage III and is moving towards clinical implementation (Fig. 4).

#### Breast and gynaecological oncology

In vivo targeting of primary breast cancer was performed with 4.5 mg bevacizumab-800CW (Table 1) administered intravenously. Back-table fluorescence imaging showed positive margins in histologically proven tumours. Ex vivo analysis demonstrated significantly higher mean fluorescence intensities of malignant tissue than surrounding normal tissue. Nevertheless, the fluorescence intensities were too low to be detected intraoperatively [76]. Another study investigating bevacizumab-800CW aimed to evaluate a standardized analysis of fluorescence images after in vivo tracer administration, called fSTREAM, based on colour images, fluorescence images, haematoxylin and eosin (H/E) microscopy slices and the pathologist’s demarcation border between malignant and normal tissue. Fluorescent signal intensity was related to tumour aggressiveness as proven by histology, resulting in 98% sensitivity and 79% specificity. fSTREAM has the capacity to guide a normalized threshold for fluorescence intensity and thus to distinguish between malignant and normal tissue. However, thresholds can differ between different tumour types and tracers [77]. Furthermore, an 88% increase in intraoperative detection rate of malignant tissue was demonstrated by retrospective histology as compared to intraoperative fluorescence signals in a 25 mg bevacizumab-800CW group [34]. Furthermore, in and ex vivo targeting was performed with the protease activatable fluorescent agent LUM015 to assess the cavity wall intraoperatively for residual tumour. The results were correlated with the histopathology of excised specimens. A total of 45 patients undergoing surgery were included for invasive ductal, lobular cancers and intraductal cancers. The sensitivity for tumour detection was 84% among all imaged surfaces and 100% sensitivity in the final cavity margin. Thus, 2 out of 8 patients (25%), with positive margins after surgery, were spared a second surgery, because additional tissue was excised at the place where a signal of LUM015 was detected [78]. Overall, it can be concluded that optical fluorescence imaging is a promising technique in the demarcation of breast cancer, which is further supported by a study that investigated a standardized analysis protocol, enhancing its potential clinical use. Because most study designs were based on dose escalation and only one calculated sensitivity and specificity, the current status of this subspeciality was deemed as stage II, with an outlook towards stage III (Fig. 4).

Gynaecological cancers were investigated with folate-FITC, which was in fact the first-in-human trial worldwide using a targeted fluorescent tracer, and OTL38 (Table 1), targeting the folate receptor alpha (FRα). In ovarian cancer patients, a dose of 0.3 mg/kg folate-FITC intravenously resulted in clear fluorescent signals, whereas no signals were observed in a patient with malignant tumour without FRα expression and in all benign tumours [79]. Metastases as small as < 1 mm were correctly identified with folate-FITC, and the number of detected tumour deposits increased significantly when fluorescence imaging was used by the surgeons (median 34 with fluorescence imaging compared to median 7 only white light) [80]. Furthermore, an optimal dose of 0.0125 mg/kg of OTL38 was determined in ovarian cancer patients, reaching 29% additional detection of malignant lesions. Fluorescence could be seen up to 1 cm beneath the tissue surface, allowing for increased malignant tissue detection as compared to white light [80].

OTL38 was also evaluated in endometrial cancer patients. After a 0.0125 mg/kg dose, an average sixfold increase of fluorescence in the tumours was measured. Moreover, 25 metastases were identified and excised, of which 19 were histologically proven malignant. All of these lesions exhibited fluorescent signals. One of these metastases was identified because of intraoperative fluorescence imaging, altering the surgical plan. Seventeen false-positive fluorescent lymph nodes were found, resulting in 100% sensitivity, 70% specificity and 48% positive predictive value [81]. Finally, in breast cancer, a new tracer, LS301, is used in patients undergoing partial mastectomy and sentinel lymph node biopsy for intraoperative margin assessment (NCT02807597). Optical fluorescence imaging in gynaecological cancers has already had an impact intraoperatively by altering the surgical plan in one study, and therefore, it has reached end-stage II clinical implementation (Fig. 4).

#### Neuro-oncology

Incomplete resection is a major problem in the transsphenoidal removal of pituitary adenomas, resulting in higher recurrence rates. Prospective cohort studies showed that a pre-operative injection of OTL38 was able to aid in the visualization of non-functional (NF) pituitary adenomas. OTL38 was able to provide 100% sensitivity and specificity in predicting resection margins in FRα-positive NF adenomas with higher specificity compared to ICG and the surgeon’s evaluation alone [82–84].

Similar to pituitary adenomas, complete resection of glioblastoma multiforme (GBM) is also challenging [85]. A feasibility study in GBM patients showed that PET and NIRF dual-modality imaging with ^68^ Ga-800CW-BBN targeted imaging via the gastrin-releasing peptide receptor (GRPR) achieved pre- and intraoperative imaging with excellent correlation. Compared to pathology, fluorescence-guided resection had a sensitivity and specificity of 94% and 100% respectively, resulting in a progression free survival at 6 months (PFS-6) of 80% in newly diagnosed GBM patients, compared to 46% in cases where non-targeted fluorophore precursors, like 5-ALA, were used [85]. A different study, where three patients received systemically administered cetuximab-800CW, proved feasibility of intraoperative visualization of GBM by NIRF imaging [86].

Tozuleristide (BLZ-100), a fluorescent tracer composed of a peptide derived from chlorotoxin (CTX) and ICG (Table 1), selectively binds to neoplastic tissue like glial tumours. Dose-escalation studies revealed that BLZ-100 was well tolerated and has potential to aid in resection of tumours in adult and in paediatric populations. BLZ-100 had lower autofluorescence and better tissue penetration compared to conventional fluorophore precursors such as 5-ALA [87, 88].

A recent study evaluated Ac-Lys^0^(800CW)Tyr^3^-ocreotate (800CW-TATE) (Table 1), targeting somatostatin receptor subtype 2 (SSTR_2_), as a potential tracer in fluorescence-guided surgery of meningiomas. Binding properties of 800CW-TATE to SSTR_2_ were tested ex vivo on ten frozen meningioma samples. Fluorescence showed a positive trend with SSTR_2_ expression and therefore facilitated in distinction between meningioma and dura mater tissue in all meningioma types [89]. Ongoing trials include the investigation of bevacizumab-800CW for intraoperative detection of pituitary neuroendocrine tumours (NCT04212793) and the intraoperative use of demeclocycline fluorescence for delineation of brain tumours (NCT02740933). Lastly, the tracer ABY-029 is used in a feasibility fluorescence imaging study of recurrent gliomas (NCT02901925).

As the majority of studies with OTL38 showed that detection of margins with high sensitivity and specificity was possible, it can be concluded that the field of targeted imaging of neuro-oncology is in stage II (Fig. 4).

#### Pulmonary oncology

Pulmonary adenocarcinomas, due to their high expression of FRα, can be targeted with folate-FITC or OTL38 (Table 1). Multiple initial feasibility studies showed that folate-FITC correctly identifies FRα-expressing tumours. Nevertheless, low signal penetration limited intraoperative imaging to subpleural tumours [90, 91]. Studies with the NIR FRα-targeting tracer OTL38 showed that it accumulated in all pre-operatively identified lesions. Nonetheless, no tumour deeper than 2 cm could be detected with fluorescence imaging in situ. Furthermore, NIR fluorescence imaging with OTL38 identified nine additional lesions in 50 patients that were unidentified by pre-operative 18-fluorodeoxyglucose PET/CT imaging. Moreover, NIRF identified 56 out of 59 nodules identified by PET/CT. For this reason, combining optical fluorescence imaging with PET/CT may result in superior oncologic outcomes [92, 93].

The applicability of optical fluorescence imaging with OTL38 in pulmonary squamous cell carcinoma (PSSC) was also evaluated. Clinical trials with OTL38 revealed intraoperative fluorescence of nodules larger than 1.1 cm and consequently prevented conversion to thoracotomy [94]. Additionally, in situ or back-table NIRF localization identified 20 out of 21 ground-glass opacities (GGO’s) altering care in 9 out of 20 subjects. Video-assisted thoracic surgery (VATS) located 10 out of 21 nodules, compared to the 15 out of 21 that were localized by NIRF imaging. Furthermore, margins assessed by NIRF imaging were similar to those assessed by pathology. NIRF imaging of tumours deeper than 1.5 cm from the pleural surface was still limited by impaired depth detection [95]. In this field, a trial with SGM-101 to detect lung metastases intraoperatively in colorectal cancers patients is currently being performed (NCT04737213).

Optical fluorescence imaging with OTL38 altered care for several patients, but since only a limited number of studies is performed, the field of pulmonary oncology is presently in stage III (Fig. 4).

#### Genitourinary oncology

Clear cell renal cell carcinomas (ccRCC) highly express carbonic anhydrase IX, targeted by girentuximab due to the overexpression of hypoxia-inducible factor-1 alpha (HIF-1α) as these tumours are fast growing and highly hypoxic. Dual-labelled ^111^In-DOTA-girentuximab-800CW was investigated in ccRCC both in vivo and ex vivo in a recently published clinical feasibility study. A large variation of antibody accumulation was observed between different tumours. Furthermore, radionuclide imaging was critical for intraoperative tumour localization as overlying fat, with its inherent increased scattering properties for light, prevented accurate localization of the fluorescent signal. Ex vivo NIRF imaging did correctly identify one additional positive surgical margin [96, 97].

A pH-sensitive tracer coupled to ICG, ICGpHLIP, was studied in urothelial carcinoma. Ex vivo instillation with this tracer accurately targeted malignant lesions in the bladder with 97% sensitivity. However, ICGpHLIP also targeted necrotic and previously treated tissue, decreasing the sensitivity to 80% [98]. Both studies proved feasibility, and therefore, this subcategory of cancers is classified as stage I (Fig. 4).

#### Soft tissue oncology

One feasibility study investigated bevacizumab-800CW in patients diagnosed with soft tissue sarcoma. An optimal dose of 10 mg administered intravenously was established. All tumour margins of the excised specimens were correctly identified with fluorescence imaging. Solid and cellular tumour masses were all easily detected, whereas border zones with more scattered malignant tissue could not be individually identified with fluorescence. False-positive fluorescent signals were observed in areas with high macrophage content, possibly due to peri-tumoral inflammation with influx of activated macrophages or inflammation induced by neoadjuvant radiotherapy resulting in increased angiogenesis [99]. Furthermore, a phase 0 study investigated ABY-029, an affibody targeting EGFR coupled to 800CW, for the resection of sarcomas. The shorter plasma half-life of affibodies compared to antibodies allows for intravenous injection 1–3 h prior to surgery compared to days [100]. This subcategory of cancer imaging was consequently deemed as stage I regarding clinical implementation (Fig. 4).

### Cardiovascular disease

We included six study reports that investigated targeted optical fluorescent imaging in carotid atherosclerosis, a highly prevalent disease which is responsible for ~ 25% of all ischemic stroke cases [101]. Initial incubation studies with clinical samples have been executed with smart activatable GLP-produced fluorescent tracer MMPSense^TM^680. This type of tracer consists of fluorophore and quencher molecules connected by peptide or oligonucleotide linkers [57, 102]. MMPSense^TM^680 emits light upon cleavage of these linkers by proteases excreted by activated macrophages which play an important role in the pathogenesis of vulnerable plaques and as such can be imaged with fluorescence and MSOT imaging (Table 1). MSOT imaging allowed for an accurate depiction of ex vivo atherosclerotic plaque morphology and activated MMPSense ^TM^680 tracer localization in three dimensions with high resolution. Single-wavelength two-dimensional image reconstruction of the MSOT device took about 30 s per image, while the creation of consecutive three-dimensional (3D) images with multiple wavelengths required approximately 20 min in total [102]. A second study investigated the tracer with ex vivo NIRF imaging, demonstrating a six- to sevenfold increase in fluorescent signals compared to the measured autofluorescence [103].

Folate receptor beta (FRβ), expressed by activated macrophages, was visualized ex vivo with folate-FITC and NIRF imaging [104]. A significant difference between background fluorescent signals after incubation with folate-FITC was observed, and clear hot and cold spots (i.e. areas displaying high uptake and low uptake, respectively) could be distinguished. Three studies focussed on the angiogenic pathway related to the vulnerable plaque, namely bevacizumab-800CW targeting VEGF-A (two studies) and scVEGF/Cy5.5 targeting the VEGF-receptor (one study) (Table 2) [105–107]. Bevacizumab-800CW was investigated with ex vivo NIRF imaging, showing a clear uptake in plaques retrieved from patients with recent ischemic events, whereas plaques retrieved from older ischemic events did not display strong fluorescent signals. However, no statistical analysis could be performed due to the small sample size [106]. A subsequent study investigated the utility of MSOT in five patients that received a low-intravenous dose of bevacizumab-800CW. Although the bevacizumab-800CW signal could not be detected with MSOT, in vivo and ex vivo NIRF imaging of the excised plaques correlated with histopathology [107]. Plaques incubated with scVEGF/Cy5.5 were imaged ex vivo [105]. Clear hot and cold spots could be identified; fluorescent signal measurements significantly increased in hot spots as opposed to cold spots. Total tracer binding occurred comparably in both symptomatic and asymptomatic plaques.

In summary, optical fluorescence imaging in the field of cardiovascular diseases has been investigated mainly in ex vivo proof of concept studies. No studies were included that evaluated a systemic clinical dose of an optical tracer with subsequent imaging. For this reason, this field is in end-stage I (Fig. 4).

### Infectious disease

Optical fluorescence imaging can provide an alternative bedside methodology for identifying bacterial infections in situ. A study in intensive care and bronchiectasis patients utilized optical endomicroscopy, known as confocal laser endomicroscopy (CLE) (Table 1) and topical administration of the lipid A-targeting antimicrobial peptide polymyxin labelled with the fluorescent 7-nitrobenz-2-oxa-1,3-diazole (PMX-NBD) (Table 1) to visualize pulmonary Gram-negative bacterial infections in the distal lung/alveoli. At this moment, however, the clinical implementation of this approach is limited by the lack of extensive diagnostic accuracy studies and fluorescent probes that were tested in situ or ex vivo for targeting polymicrobial or Gram-positive bacterial infections [108].

Optical fluorescence imaging can also be used to visualize infections ex vivo. P2&3TT is a smart activatable oligonucleotide-based probe that carries fluorescein amidite on the 5′-end and the ZEN and Iowa Black RQ quenchers on the 3′-end. The probe is activated upon cleavage by the *Staphylococcus aureus* (SA) micrococcal nuclease (Table 1). P2&3TT can be used for fast ex vivo detection of SA in blood cultures with a 10^4^-fold higher sensitivity compared to conventional diagnostic culturing methods. This method may assist as a complementary tool in the diagnosis of *S. aureus* bacteraemia [109].

Both of the aforementioned studies are proof of concept, and therefore, the clinical use of optical fluorescence imaging for infectious disease is currently still in stage I (Fig. 4).

## Discussion

This review provides an overview of all relevant literature on the topic of clinical targeted optical fluorescent imaging with the aim to chart the latest developments in the various clinical disciplines that have started to make use of optical fluorescence imaging techniques towards routine clinical application in case of value. Especially in the field of gastroenterology and oncology (i.e. head and neck cancer), the intraoperative use of fluorescence imaging has a high proven impact on future clinical decision-making (end-stage III). Other areas of oncology are presently in stage III (pulmonary, neurological), stage II (gynaecological and breast) and stage I (soft tissue and genitourinary). Despite the fact that the development of optical fluorescence imaging in cardiovascular (end-stage I) and infectious diseases (stage I) is still less advanced, the concept is gaining momentum in these specialties as well, promoting the potential broad clinical implementation of this imaging approach.

So far, the most mature areas concern the targeted optical fluorescent imaging of GI-tract and head and neck oncology (end-stage III). The reason for this resides presumably in their readily accessible location for imaging with white light. In the fields of cardiovascular and infectious diseases, feasibility studies still dominate. However, it is anticipated that in both fields, further development will depend on the expansion of investigated clinical applications. With respect to infectious disease, for example, extensive pre-clinical and post-mortem studies that utilize the targeted vancomycin-IRDye800CW conjugate have already been conducted [110, 111]. These studies are likely to open up new clinical possibilities for targeting Gram-positive bacterial infections in the near future once this tracer is GMP produced and evaluated in phase I/II clinical safety/feasibility studies.

While a variety of fluorescent optical antibody- or small molecule-based, smart activatable and pH-based tracers have been applied to date, we noticed that only a limited repertoire of fluorescent labels is currently in use. The IRDye800CW, which was mostly used for labelling antibodies, and S0456 (bound to OTL38) account for approximately 75% of the applications of the investigated fluorophores. Relatively new are dual-labelled tracers (i.e. nuclear and fluorescent), pH-based and smart activatable tracers, which increase the utility of fluorescent imaging. Furthermore, the evolution of total body PET/CT may instigate significant changes in the need for fluorescent compounds, as the radiation burden will be lower while PET-detection sensitivity has increased and follow-up times for treatment response measures may increase [112]. Dual-labelled probes contain a mixture of both radioactive and fluorescent labels, facilitating pre-operative (deep) target tissue visualization with conventional techniques, such as PET/CT, and intraoperative visualization with NIRF imaging [85, 96, 97]. Novel pH-based tracers, such as ONM-100 and ICG-pHLIP, which are activated in acidic conditions, may help to circumvent the need for specific receptor or antigen expression [58, 98]. Previous research has also shown the feasibility of such pH-responsive tracers in cancer treatment as opposed to only tumour visualization, which further highlights the versatility of such tracers in a theranostic approach [113].

Two types of activatable or “smart” probes were described in this work: MMPSense^TM^680 and P2&3TT. Activation occurs through cleavage of the peptide/oligonucleotide linkers by specific enzymes, which will separate the fluorophore from the quencher. Consequently, the probe is switched “on” and emits a fluorescent signal. This mode of action is promising for future research and clinical implementation, as it allows for increased contrast and sensitivity for specific molecular targets and, additionally, it minimizes background signals compared to tracers that have an always “on” state [57].

Today, optical fluorescent imaging in general is mainly limited by the low tissue penetration of fluorescent signals. The fluorescent light emitted by NIRF agents has a somewhat better tissue penetration, but the problem persists for deeper situated or covered target tissue [92]. Applying photoacoustic imaging techniques, such as MSOT, could provide a solution for this problem by increasing image resolution and imaging depths (± 5 cm) compared to NIRF optical imaging methods (< 2 cm) [56, 114]. For this reason, we anticipate that future research on MSOT imaging and targeted MSOT-tailored imaging agents may contribute to clinical translation. Furthermore, the development of implantable and biodegradable optical waveguides, which can assist in the delivery of light, also provides a promising platform to overcome the hurdles imposed by tissue turbidity [115].

One other major challenge for (targeted) clinical optical fluorescent imaging in making the final step towards the clinic will be the design of standardized image acquisition, quantification, validation and reporting methods. The lack of standardized protocols may have the consequence that future study results will not be comparable and/or reproducible due to heterogeneous outcomes and insufficient towards regulatory approval by agencies like the FDA and EMA. This may lead to the unjustified conclusion that optical fluorescent imaging applications cannot be used for specific clinical purposes, resulting in the unnecessary loss of technology with great potential for the benefit of future patients. Several ICG-guided perfusion studies, for example have attempted to predict anastomotic bowel leakage utilizing a non-standardized fluorescence imaging approach [116–118]. Consequently, these studies may lead to heterogenous results, reporting and conclusions based on heterogenous datasets potentially leading to premature conclusions that optical fluorescent ICG perfusion imaging does not significantly contribute to the intraoperative prediction of anastomotic leakage. In order to tackle such an omission, a standardization method for the non-targeted tracer ICG has recently been developed. This proof of concept study showed that the proposed and validated ICG quantification approach, coined WISQ, can aid in prediction of post-operative organ function impairment [119]. Such methodologies are required in order to make optical fluorescent imaging perfusion applications standard of care in the clinic.

During our literature searches, we only identified one study that has investigated the implementation of a standardization method for targeted tracers: fSTREAM. This analytical method combines fluorescent and white-light data with histology to quantify fluorescent signals and determine a normalized threshold for breast cancer tissue as compared to healthy tissue. fSTREAM was validated for the spatial mapping of fluorescent signals in breast cancer and it holds promise for the demarcation of other tumour types [77]. We foresee that reproducibility, widespread clinical implementation and subsequent clinical decision-making based on optical fluorescent imaging will depend on the development of such quantitative methods. Ideally, such methods will be able to combine optical fluorescent imaging data with more conventional imaging data produced with approved imaging modalities like CT, MRI, SPECT and PET/CT when needed. One of the concepts that we believe could add to this standardization is the creation of dedicated optical fluorescence-guided operating theatres for the use of standardized fluorescence-guided surgery, as demonstrated for GI-tract cancers [61]. Such operating theatres could facilitate efficient and standardized intraoperative and back-table specimen imaging. Another concept that could help clinical translations is the standardization of imaging equipment. Currently, due to the lack of standardization, combined drug-device registration is required leading to additional cost and complexity [120]. Furthermore, based on the previous observations and the different tracer types that have so far been explored, we anticipate that there will be an increasing need for tumour agnostic tracers, which can be used in, e.g. multiple types of cancers and multimodal tracers, which will offer multiple simultaneous imaging opportunities for clinicians in terms of visualizing target tissue and the combined use of different imaging modalities. Other directions for future research are listed in (Table 3).

**Table 3:** Suggestions for future research on optical fluorescence imaging

Need	Solution
Guideline development based on large studies	Performing pivotal and statistically well-powered phase 3 clinical studies to provide more evidence of targeted optical fluorescent imaging on clinical decision-making and patient outcomes. This will support the set-up of guidelines for appropriate use
Combining optoacoustic imaging and targeted tracers	Investigate the options regarding application of optoacoustic imaging in combination with available targeted tracers for increasing the signal and more specific imaging [56]
Therapeutic fluorophore development	Designing and validating new fluorophores with properties suitable for medical purposes, e.g. appropriate size, positive effects on targeting agents and beneficial pharmacokinetic properties [113]
Combination with light-activated therapeutics	Focus on development of light-controlled therapeutic modalities, such as photodynamic therapy and photo pharmacology, and their combination with optical fluorescence imaging as a theranostic modality [124]
Multimodal tracer development	Design, synthesis and evaluation of multimodal tracers that allow combination of optical fluorescence imaging with other imaging techniques (PET/CT, SPECT, MRI, CT and ultrasound). This offers the opportunity of combined pre-operative and intraoperative imaging. This also facilitates integration of optical fluorescent imaging with already clinically established imaging modalities [73, 85, 96, 97]
“True” quantification methods	Develop standardization methods which can truly quantify and validate optical fluorescence imaging [77, 119]

Importantly, optical fluorescent imaging has already shown its potential to aid in clinical decision-making. Gastrointestinal and head and neck cancers are currently taking a lead in the field of targeted fluorescent imaging. Although optical fluorescence imaging is still in its early clinical translational phase, it offers an innovative range of attractive possibilities concerning fluorescence-guided surgery and disease monitoring, for which it may become an important component of the standard care package in the near future. For example, sentinel lymph node biopsy (SLNB) using ICG has already shown to be as accurate as SLNB using a radioactive tracer, demonstrating the clinical potential of fluorescence imaging techniques [121–123]. The value of optical fluorescent imaging will be even more evident once it has been complemented and enriched with generally accepted standardization and quantification protocols and ultimately providing benefit for the individual patients based on accurate, reproducible and reliable datasets and execution of standardized imaging protocols and quantification methods for each individual imaging session, similarly as is presently the case for the conventional imaging modalities.

This review presents an overview of the field of targeted optical fluorescent imaging, the current status of targeted optical fluorescent imaging and the directions for future research. Despite the systematic methodology that was implemented, a few limitations should be mentioned. First of all, a relatively large number of articles were included by reference screening. This is partially explained by the diverse terminology used in optical fluorescent imaging. Second, the usage of artificial intelligence-based software, such as ASReview, may result in relevant articles being missed, because not all search results are explicitly read and screened for eligibility. Other drawbacks of ASReview are the inability to evaluate the system’s error rate and the absence of empirical benchmarks of its performance [40]. Third, we had to narrow down the scope of this review to targeted optical fluorescence imaging due to the many articles available about non-targeted fluorescence optical imaging. However, this allowed us to focus on the novel methodologies of targeted optical fluorescence imaging.

In conclusion, the field of clinical targeted optical fluorescent imaging is rapidly evolving and expanding, especially in the context of oncology. Nonetheless, this imaging methodology still needs to overcome some major hurdles before it can be part of the standard of care in many clinical applications. Intensive clinical collaboration between nuclear medicine physicians, chemists, pharmacists, physicists, medical specialists and the regulatory agencies and reimbursement policy-makers will be of critical importance for a wider clinical implementation of targeted optical fluorescence imaging. For this reason, it is advisable to establish a clinical optical fluorescent imaging task force in which the aforementioned specialisms collaborate to bring this important field to maturity and clinical standard of care.

## Supplementary Information

Below is the link to the electronic supplementary material.

Supplementary file1 (DOCX 16 kb)

## Data Availability

Not applicable.
