# Supplementary material for: Targeted optical fluorescence imaging: a meta-narrative review and future perspectives
Source: Eur J Nucl Med Mol Imaging. 2021 Oct 11;48(13):4272–92. doi: 10.1007/s00259-021-05504-y (PMC8566445; doi:10.1007/s00259-021-05504-y)
Supplement: Supplementary file 1 — Supplementary file1 (DOCX 16 kb) [file 259_2021_5504_MOESM1_ESM.docx]

**Article title:** Targeted optical imaging: a meta-narrative review and future perspectives

**Journal name:** European Journal of Nuclear Medicine and Molecular Imaging

**Authors:** HM Schouw^1*^, LA Huisman^1*^, YF Janssen^1^, RHJA Slart^2,3^, RJH Borra^2,4^, ATM Willemsen^2^, AH Brouwers^2^, JM van Dijl^5^, RA Dierckx^2,6^, GM van Dam^2^, W Szymanski^4^,

HH Boersma^2,7^ ª, S Kruijff^1,2^ ª

**Affiliations:**

1. Department of Surgery, University of Groningen, University Medical Centre Groningen, Groningen, The Netherlands
2. Department of Nuclear Medicine and Molecular Imaging, University of Groningen, University Medical Centre Groningen, Groningen, The Netherlands
3. Department of Biomedical Photonic Imaging, Faculty of Science and Technology, University of Twente, The Netherlands.
4. Department of Radiology, University of Groningen, University Medical Centre Groningen, Groningen, The Netherlands
5. Department of Medical Microbiology, University of Groningen, University Medical Centre Groningen, Groningen, The Netherlands
6. Department of Diagnostic Sciences, Ghent University Faculty of Medicine and Health Sciences, Gent, Belgium
7. Department of Clinical Pharmacy and Pharmacology, University of Groningen, University Medical Centre of Groningen, Groningen, The Netherlands

* Authors share first authorship and contributed equally to this work

ª Authors share last authorship and contributed equally to this work

**Corresponding author:**S Kruijff, [s.kruijff@umcg.nl](mailto:s.kruijff@umcg.nl)

**Supplement I:** PubMed search string.

|  | **Optical imaging** | **Oncology** | **Cardiovasculair** | **Infectie/inflammatie** |
| --- | --- | --- | --- | --- |
| **MeSH** | “Optical imaging”[Mesh]  **AND**  “Fluorescence”[Mesh]  OR  “Fluorescent Dyes”[Mesh] | "Neoplasms/diagnostic imaging"[Mesh] | “Cardiovascular  Diseases”[Mesh] | "Infections"[Mesh]  OR  "Inflammation"[Mesh] |
| **Title/Abstract** | “Optical imaging[tiab]  OR  “Fluorescence imaging[tiab]  **AND**  “Fluorescent Dy*”[tiab]  OR  “Fluorescence Agen*”[tiab] OR  “Fluorescent Agen*”[tiab] OR  Fluorochrom*[tiab]  OR  “Fluorescent Prob*”[tiab]  OR  “Fluorogenic substan*”[tiab] OR  “Fluorescent antibod*”[tiab] OR  “Fluorescent tracer*”[tiab] OR  “Near-infrared”[tiab]  OR  “Near infrared”[tiab]  OR  “Fluorescence-labelled antibod*”[tiab] | “Neoplas*”[tiab]  OR  “Oncolog*”[tiab]  OR  “Cancer*”[tiab]  OR  “Tumor*”[tiab]  OR  “Tumour*”[tiab]  OR  “Malignan*”[tiab]  OR  “Metasta*”[tiab] | “Cardiovascular diseas*”[tiab]  OR  “Heart diseas*”[tiab]  OR  “Vascular”[tiab]  OR  “Aneurysm*”[tiab]  OR  “Atherosclero*”[tiab]  OR  “Cerebrovascular”[tiab]  OR  “Myocard*”[tiab]) | “Infectio*”[tiab]  OR  “Infestatio*”[tiab]  OR  “Iinflammat*”[tiab]  OR  “Inflamed”[tiab]) |
